# Concomitant intestinal malrotation and Crohn’s disease: a rare and surgically challenging anomaly

**DOI:** 10.1093/jscr/rjac152

**Published:** 2022-04-12

**Authors:** Jacob Silverman, Benjamin Salwen, Idan Goren, Ian White

**Affiliations:** Department of Medicine, Tel Aviv University, Tel Aviv, Israel; Department of Medicine, Tel Aviv University, Tel Aviv, Israel; IBD Center, Division of Gastroenterology, Rabin Medical Center, Petah Tikva, Israel; Colorectal Surgery, Department of Surgery, Rabin Medical Center, Petah Tikva, Israel

## Abstract

Crohn’s disease (CD) is a chronic inflammatory bowel disease characterized by transmural inflammation occurring anywhere along the gastrointestinal tract. Intestinal malrotation is an embryological error resulting in an abnormal gut anatomy. Although these two conditions rarely present concurrently, it is important to identify their presence, which is challenging due to their nonspecific, overlapping symptoms. Here, we present two patients with concomitant CD and intestinal malrotation. Both patients’ conditions required surgical intervention, which was complicated due to their unique anatomy. Clinicians should be aware of the potential pit-falls that may occur due to the anomaly and thus require a full understanding of the anatomy.

## INTRODUCTION

Intestinal malrotation is a result of an error in the embryonic rotation of the gut. Normal intestinal anatomy develops as a result of 270° of counterclockwise rotation [1]. This process can be disrupted at any point during development, resulting in abnormal gut anatomy and typically presents in infancy with several nonspecific symptoms [2].

Crohn’s disease (CD) has an incidence of 3–20 cases per 100 000. Its incidence has been steadily increasing, globally [3]. Although the exact pathogenesis of CD is not fully understood, the disease presents with chronic transmural inflammation that may occur anywhere in the gastrointestinal tract and is characterized by skip lesions and granulomatous inflammation on biopsy [4]. This inflammation can result in strictures, fistulas and perforation often necessitating surgery.

The combination of intestinal malrotation and CD is quite rare, with three cases previously described in the literature. Here, we present two cases of patients with previously diagnosed intestinal malrotation and concurrent CD who underwent surgery for CD complications at our institution and are presented in order to add to the global experience of this unusual condition.

## CASE REPORT

### Patient 1

A 40-year-old male patient with a history of CD presented with increasing flares of abdominal pain and weight loss of 8 kg in the previous year. The patient has a known history of intestinal malrotation diagnosed at age 12 and a chronic small bowel obstruction. As a child he underwent a Ladd’s procedure.

**Figure 1:**
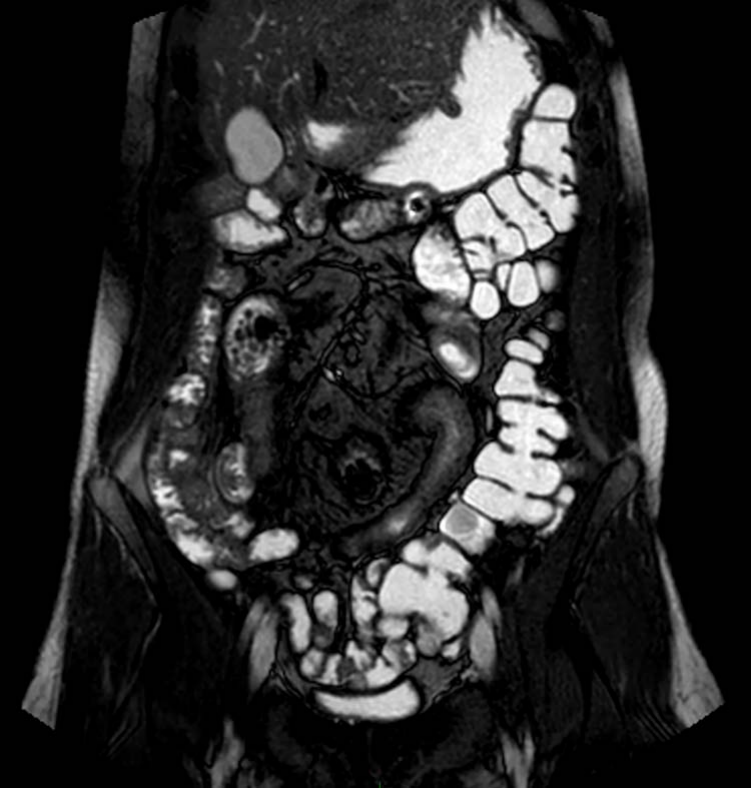
MRI enterography depicting intestinal malrotation and ileal loops in the central abdomen dilated up to 5 cm, consistent with malrotation and a small bowel obstruction.

Magnetic resonance imaging (MRI) enterography conducted a year prior to the current admission demonstrated the entire colon was found to the left of the abdominal midline (Fig. 1). MRI demonstrated ileal loops in the central abdomen, a thickened bowel wall with dilated sections ~5 cm in diameter. These findings were consistent with malrotation of the bowel and small bowel obstruction.

Due to history of CD, malrotation and chronic small bowel obstruction the decision was made to resect the small intestine. Surgical exploration was performed to alleviate obstruction and for failure of response to steroids, biological treatment and exclusive enteral nutrition.

### Surgical course

**Figure 2:**
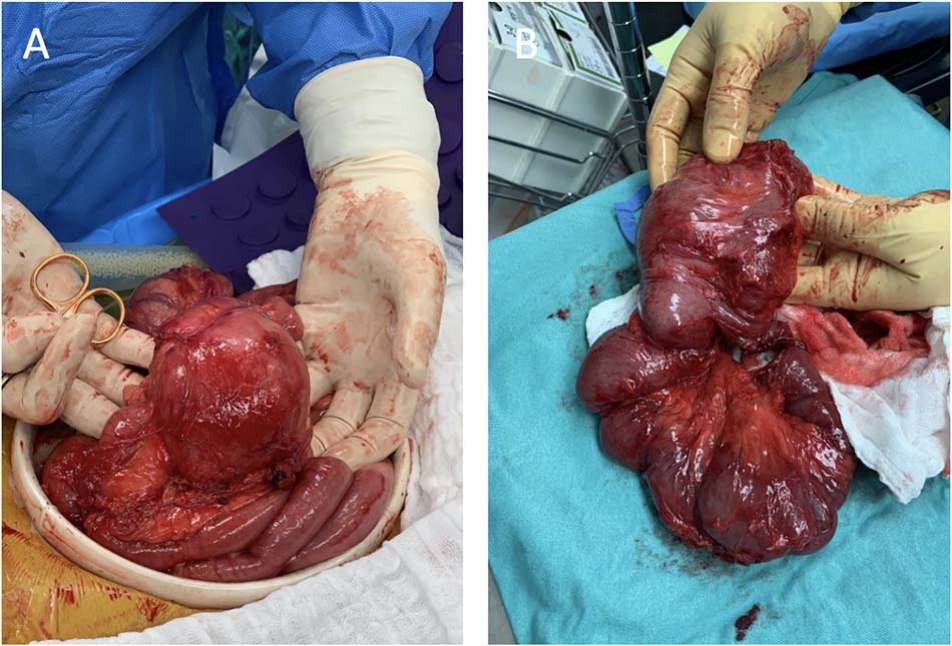
Intraoperative images depicting malrotation of the inflamed intestine (**A**) and a 35-cm segment of the distal terminal ileum that was resected (**B**).

The surgery commenced with laparoscopy under general anesthesia. Many adhesions from previous surgeries and malrotation were noted, with the colon found in the left side of the abdominal cavity and the small intestine on the right. Due to the degree of adhesions and malrotation, the procedure was converted to an open approach through a midline incision. The section of the ileum causing the obstruction was visualized in the distal ileum, which included severe stenosis, with nearly 8-cm dilated small bowel proximal to the stenosis (Fig. 2).

A Heineke–Mikulicz strictureplasty was performed on the proximal stricture. A length of 35 cm of the distal terminal ileum was resected and a side-to-side anastomosis to the proximal small bowel was performed.

The immediate postoperative course was uneventful, and the patient was discharged 4 days following the surgery. The patient was readmitted 4 days after discharge with shortness of breath and fever. He was diagnosed with nosocomial pneumonia and was treated with piperacillin-tazobactam. Bowel function and alimentation returned to baseline.

### Patient 2

A 26-year-old male presented 6 months prior to his surgery with abdominal pain, fever, non-bloody diarrhea and 13 kg of weight loss. He was given a course of antibiotics and underwent a colonoscopy where ileo-colonic disease was discovered. He was diagnosed with CD and malrotation was incidentally discovered at this time.

Two months prior to surgery and before initiating CD treatment, the patient was hospitalized. A computed tomography (CT) demonstrated a 30-cm region of marked thickening in the terminal ileum, an adjacent multilocular abscess measuring 4.3 × 3 × 2.5 cm and intestinal malrotation. The patient was managed conservatively with antibiotics and total parenteral nutrition (TPN). Drainage could not be performed due to the abscess location.

**Figure 3:**
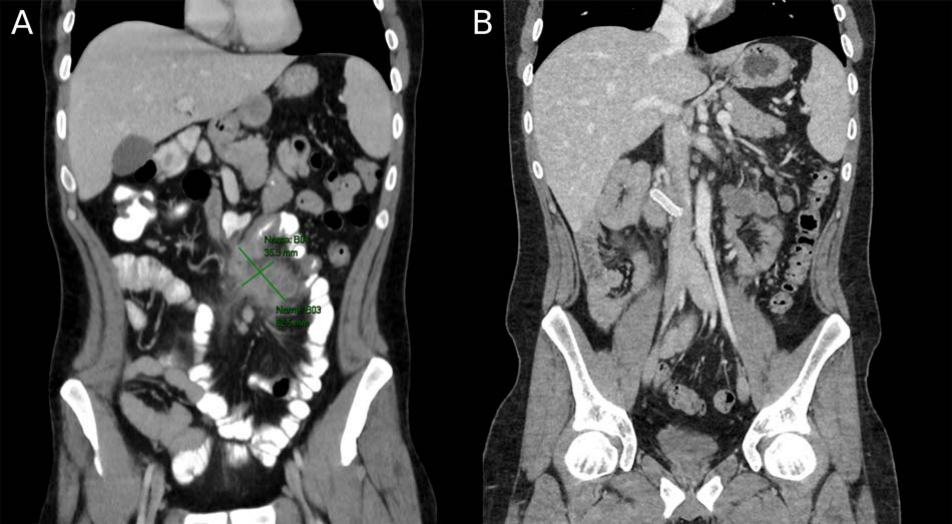
(**A**) Preoperative image depicting intestinal malrotation and an abdominal abscess measuring 62.5 × 35.3 mm. (**B**) Postoperative image depicting a Gore-tex graft between the superior mesenteric vein and inferior vena cava.

A follow up colonoscopy performed 1 month after antibiotic treatment demonstrated cobblestone mucosa along with a severe stenosis in the terminal ileum and a small bowel fistula. In addition, an abdominal CT showed a 10-cm section of bowel wall thickening in the terminal ileum and remnants of the abscess (Fig. 3). Ileocecectomy was electively planned due to the severity of disease and persistence of symptoms.

Surgical exploration via laparoscopy was performed. Intestinal malrotation was identified in addition to a 30-cm section of inflamed bowel in the terminal ileum. The laparoscopy was converted to open laparotomy as the blood vessels could not be correctly identified and the abscess was adhesing the bowel wall to the mesentery. Despite laparotomy, resection of the terminal ileum along with the cecum was difficult and the superior mesenteric vein (SMV) could not be identified. As a result of subsequent congestion of the small bowel, the proximal SMV was identified without continuation to the small bowel mesentery. It appeared that the congestion was due to dissection of the ileocolic veins disrupting venous drainage from the small bowel. The superior mesenteric artery was normal. The SMV root was then anastomosed to the inferior vena cava with a Gore-tex graft. Small bowel congestion improved after vascular reconstruction, but mild congestion persisted. The abdomen was closed and a second look surgery within 24 h was planned. The following day repeat laparotomy revealed a viable small and large intestine, and an end ileostomy was performed. The large bowel remnant was left closed within the abdomen.

The immediate postoperative course included admission to the intensive care unit, where he remained intubated while undergoing intense postoperative monitoring due to the vascular aspect of the surgery. Postoperative treatment included 9 days of piperacillin-tazobactam on recommendation from the vascular team and 11 days of TPN. The patient was discharged 15 days postoperatively in good condition with long-term anticoagulation due to the Gore-tex graft.

## DISCUSSION

Intestinal malrotation and concurrent CD is exceedingly rare; with only three cases reported in the literature (Table 1). The first case published in 1954 describes a patient with midgut malrotation found incidentally during laparotomy for partial intestinal resection due to CD [5]. Two subsequent case reports of CD and malrotation have been published in 2011 and 2014 [6, 7]. In both these cases malrotation was noticed on imaging prior to the surgery.

**Table 1:** Summary of document cases of concomitant Crohn’s disease and Malrotation

Citation	Patients	Age	Gender	Presenting symptoms	Diagnosis of malrotation concurrent with Crohn’s diagnosis	Intervention	Adverse events
Fieber SS, 1954	1	28	Male	Abdominal pain, weight loss, weakness, malaise, enlarging abdominal mass	Yes	Exploratory laparotomy	Not stated
Brown NM, 2011	1	24	Female	Nausea, emesis, abdominal pain	Yes	Ileocecectomy with primary side-to-side anastomosis	Not stated
Fiorani C, 2014	1	51	Male	Abdominal pain	No	Ileocecectomy with primary side-to-side anastomosis	None
Silverman J, 2021	2	40	Male	Abdominal pain, weight loss	No	Heineke–Mikulicz strictureplasty with ileal resection and primary side-to-side anastomosis	Nosocomial pneumonia
	26	Male	Abdominal pain, non-bloody diarrhea, weight loss	Yes	Ileocecectomy with end ileostomy	Dissection of ileocolic veins resulting in anastomosis between SMV and IVC with Gore-tex graft	

SMV, superior mesenteric vein; IVC, inferior vena cava.

Intestinal malrotation is a congenital anomaly involving malpositioning of the small bowel and colon as well as malfixation of the mesentery that frequently presents in the first months of life. Malrotation presents symptomatically in ~1 in 6000 patients, however, it is commonly asymptomatic and thus the true incidence is likely higher [1]. Adult presentation is rare accounting for 0.2–0.5% of diagnosed cases of malrotation [8].

Normal intestinal rotation begins in the fourth gestational week. The embryonic intestinal loop herniates into the umbilical cord and rotates counterclockwise 90°. In the eighth to tenth week the intestine returns to the abdominal cavity and continues to rotate another 180°, totaling 270° counterclockwise [9]. This results in normal anatomy with the duodenojejunal flexure to the left of the midline and the cecum in the right lower quadrant. Malrotation represents an error in this process, most commonly causing the cecum to be found left of midline, the duodenojejunal flexure on the right, with a narrow, malfixed mesentery and Ladd’s bands crossing the duodenum from the right towards the cecum [10]. Common associations with malrotation include trisomy 21, cardiac defects, anorectal malformation and duodenal web [11]. To date, no mechanistic association between midgut malrotation and CD are known.

The clinical presentation of malrotation can be nonspecific and difficult to diagnose, particularly with comorbid CD. Patients with malrotation can suffer from repeated acute bowel obstruction as well as less specific symptoms such as generalized abdominal cramping [12]. Due to the vague nature of these symptoms malrotation is frequently not discovered until later in life, often incidentally on imaging. The specific presentation in adulthood can include ‘left-sided’ appendicitis and midgut volvulus [10]. It is important to note especially in the case of our patients that many of the symptoms of malrotation can overlap with those of CD. It is important to be aware that multiple etiologies may account for these symptoms and both diseases may simultaneously present.

Malrotation itself is not a surgical indication, in cases such as our patients with CD, it can provide a surgical as well as endoscopic challenge. It is of note that both patients required conversion to open procedures and the second patient required vascular reconstruction, perhaps due to anatomical variation of the vascular anatomy secondary to the underlying malrotation. With these concomitant conditions, one should consider advanced imaging prior to surgical intervention. A potential option that has been shown to be efficacious in inflammatory bowel disease patients is virtual endoscopy, which can be used to create 3D models with MRI [13]. It is important to note that proper understanding of the anatomy and the use of imaging are necessary to reduce the potential complications involved in surgical intervention.

## CONCLUSION

Concomitant CD and malrotation is a rare combination. Therefore, we cannot conclude that there is a mechanistic relationship or causality between them. Although proper understanding of the anatomical anomaly based on imaging is recommended, anomalies can still be challenging to operate and are possibly at higher risk for complications.

## FUNDING

No external funding was provided for this study.

## CONFLICT OF INTEREST STATEMENT

None declared.
